# Cytologic scoring of equine exercise-induced pulmonary hemorrhage: Performance of human experts and a deep learning-based algorithm

**DOI:** 10.1177/03009858221137582

**Published:** 2022-11-17

**Authors:** Christof A. Bertram, Christian Marzahl, Alexander Bartel, Jason Stayt, Federico Bonsembiante, Janet Beeler-Marfisi, Ann K. Barton, Ginevra Brocca, Maria E. Gelain, Agnes Gläsel, Kelly du Preez, Kristina Weiler, Christiane Weissenbacher-Lang, Katharina Breininger, Marc Aubreville, Andreas Maier, Robert Klopfleisch, Jenny Hill

**Affiliations:** 1 University of Veterinary Medicine Vienna, Vienna, Austria; 2 Freie Universität Berlin, Berlin, Germany; 3 Friedrich-Alexander-Universität Erlangen-Nürnberg, Erlangen, Germany; 4 EUROIMMUN Medizinische Labordiagnostika AG, Lübeck, Germany; 5 Novavet Diagnostics, Bayswater, Western Australia; 6 University of Padova, Legnaro, Italy; 7 University of Guelph, Guelph, Ontario, Canada; 8 Justus-Liebig-Universität Giessen, Giessen, Germany; 9 University of Pretoria, Pretoria, South Africa; 10 Technische Hochschule Ingolstadt, Ingolstadt, Germany

**Keywords:** artificial intelligence, automated image analysis, bronchoalveolar lavage fluid, computational pathology, digital pathology, equine, pulmonary hemorrhage, respiratory disease, total hemosiderin score

## Abstract

Exercise-induced pulmonary hemorrhage (EIPH) is a relevant respiratory disease in sport horses, which can be diagnosed by examination of bronchoalveolar lavage fluid (BALF) cells using the total hemosiderin score (THS). The aim of this study was to evaluate the diagnostic accuracy and reproducibility of annotators and to validate a deep learning-based algorithm for the THS. Digitized cytological specimens stained for iron were prepared from 52 equine BALF samples. Ten annotators produced a THS for each slide according to published methods. The reference methods for comparing annotator’s and algorithmic performance included a ground truth dataset, the mean annotators’ THSs, and chemical iron measurements. Results of the study showed that annotators had marked interobserver variability of the THS, which was mostly due to a systematic error between annotators in grading the intracytoplasmatic hemosiderin content of individual macrophages. Regarding overall measurement error between the annotators, 87.7% of the variance could be reduced by using standardized grades based on the ground truth. The algorithm was highly consistent with the ground truth in assigning hemosiderin grades. Compared with the ground truth THS, annotators had an accuracy of diagnosing EIPH (THS of < or ≥ 75) of 75.7%, whereas, the algorithm had an accuracy of 92.3% with no relevant differences in correlation with chemical iron measurements. The results show that deep learning-based algorithms are useful for improving reproducibility and routine applicability of the THS. For THS by experts, a diagnostic uncertainty interval of 40 to 110 is proposed. THSs within this interval have insufficient reproducibility regarding the EIPH diagnosis.

Exercise-induced pulmonary hemorrhage (EIPH) in horses is a disease characterized by (repeated) hemorrhage from the lungs during high-intensity athletic activity.^16^ This disease is reported with very high prevalence in numerous breeds of sport horses.^34^ Although the underlying pathophysiological mechanisms and predisposing risk factors of EIPH are not fully understood, it has been shown that severe EIPH has a negative impact on athletic performance in horses.^11,16,17^

Following pulmonary bleeding, red blood cells (RBCs) are removed by mucociliary clearance through the upper airways or degraded to hemosiderin (iron-protein-complex derived from breakdown of hemoglobin) by alveolar macrophages. The presence and severity of EIPH can be evaluated by tracheobronchoscopic examination,^11,16^ or quantification of RBC components in respiratory tract fluid. Although there are numerous diagnostic methods with different specificities and sensitivities, noted below, a true gold standard method (such as chemical quantification of hemosiderin) is lacking.^10,12,35^

Tracheobronchoscopic evaluation of blood content in the airways shortly after strenuous exercise has been proposed as the best available method by the American College of Veterinary Internal Medicine.^16^ This method is relatively easy to perform and seems to have a very high specificity.^19^ However, sensitivity was estimated to be only 59% (many false-negative diagnoses) when compared with RBC content in respiratory fluid.^19^ Therefore, it has been proposed that a lack of tracheobronchoscopic evidence of blood cannot be used to rule out EIPH.^19,29^

Diagnosis of EIPH through examination of respiratory tract fluids has been derived from the RBC content,^19,31^ hemosiderin content in alveolar macrophages,^12,14^ or less commonly hemoglobin concentration.^31^ When compared with tracheobronchoscopy, these tests are generally assigned a higher sensitivity and many authors have recommended these as the best available diagnostic tests.^14,17,19,29,34,35^ Whereas RBC counts can only be used to diagnose a recent EIPH episode within a few hours to days, increased hemosiderin content in alveolar macrophages (ie, hemosiderophages) may reveal less recent EIPH episodes.^10^ Previous studies have found that increased numbers of hemosiderophages can be detected from 7 up to 28 days after a single event of pulmonary bleeding or experimental blood inoculation.^25,29,33^

Different cytologic, semi-quantitative scoring systems to evaluate hemosiderin content in alveolar macrophages have been proposed, which either use conventional cytological staining or specific iron stains (eg, Prussian blue) for hemosiderin.^12–14,18,30^ The most complex scoring system by Doucet and Viel grades the intracytoplasmic hemosiderin content of 300 alveolar macrophages into 5 tiers, based on the amount of blue hemosiderin pigment, using special iron stain. Scoring ranges from 0 (absence of intracytoplasmic hemosiderin) to 4 (macrophages are filled with hemosiderin). Subsequently, the total hemosiderin score (THS) is calculated per 100 cells, and can range from 0 to 400. Compared with post-exercise tracheobronchoscopy, the presence of EIPH was best predicted at a cut-off value of THS ≥ 75, with a sensitivity of 94% and a specificity of 88%.^14^ Although this scoring system is probably the most sensitive and presumably most reproducible diagnostic test currently available, it has been declared unsuitable for routine diagnostic use due to the high expenditure of human labor.^10^ Regardless of its quantitative nature, previous studies have also shown that grading hemosiderin content of individual cells based on the definition by Doucet and Viel has some interrater and intrarater inconsistencies.^20,22^

To overcome these limitations of the THS, a deep learning-based algorithm for automated image analysis has been developed by our research group.^20,23^ Automated image analysis is a field of great interest in veterinary medicine and is becoming increasingly feasible with incorporation of whole slide image (WSI) scanners into the workflow of veterinary laboratories, appropriate information technology (IT) infrastructure and computational power, and advancing artificial intelligence methods, specifically deep learning.^8,24,28,36^ However, a thorough validation of those algorithms is necessary before they can be used for routine diagnostic service or clinical research.^24,28^

The aim of the present study was to determine the interobserver variability of the THS between 10 human experts (annotators) and to validate whether the diagnosis of EIPH can benefit from the use of a deep learning-based algorithm. The performance of the algorithm was compared with the 10 annotators, a ground truth dataset, and chemical measurements. Our hypothesis was that the use of a deep learning-based algorithm allows for a more efficient THS analysis while having a high diagnostic consistency and accuracy that is at least equivalent to the annotators.

## Materials and Methods

### Study Specimens (Cytologic WSIs)

**Figure 1.**
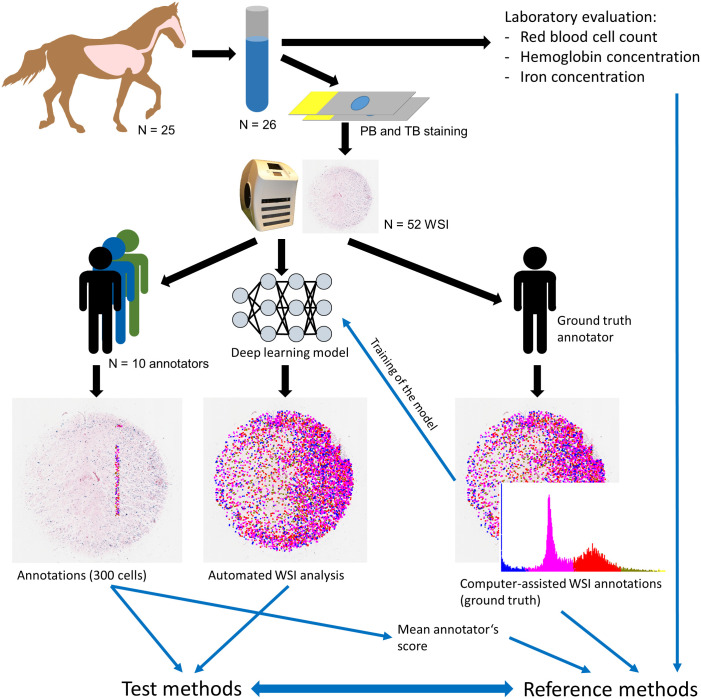
Overview of the study material and methods. Abbreviations: PB, Perl’s Prussian blue; TB: modified Turnbull’s blue (Quincke reaction); WSI, whole slide image.

For this study, 29 bronchoalveolar lavage fluid (BALF) samples from 25 horses, including 2 samples from each of 4 horses with separate BALF samples from the left and right lungs, were prospectively collected from routine diagnostic samples submitted to VetPath Laboratory Services (Ascot, Australia; Fig. 1). Twenty-eight samples were submitted for routine evaluation of EIPH and 1 case was submitted for routine evaluation of equine asthma. Use of these samples for this study was approved by the State Office of Health and Social Affairs of Berlin, Germany, approval ID: StN 011/20. Two cytological specimens per BALF sample were prepared using cytocentrifugation (CYTOPRO 7620, Wescor Inc., Logan, UT, USA) of a variable volume of BALF (depending on cellular density) at 510 × g for 3 minutes. Unstained specimens were sent to the FU Berlin, Germany, and 1 of the 2 specimens was stained with Perl’s Prussian blue and the other using a modified Turnbull’s blue, Quincke reaction, according to standard protocols.^32^ In both cytochemical staining methods, nonheme-iron reacts with the staining solution forming an insoluble blue pigment.^26^ Hemosiderin is largely composed of ferric iron (Fe^+3^); however, there is also some ferrous iron (Fe^2+^) present along the margins of hemosiderin.^27^ While Prussian blue stains iron in the ferric state, Turnbull’s blue detects Fe^2+^ and is considered less suitable to stain hemosiderophages. However, the Quincke reaction uses a pretreatment with ammonium sulfide that reduces Fe^3+^ to Fe^2+^, therefore, iron of both oxidation states is stained by the modified Turnbull’s blue reaction.^32^ Nuclear Fast Red solution was used to counterstain nuclei. Although previous studies on equine EIPH mainly used Prussian blue,^12,14^ we used both staining methods in an attempt to increase image variability which in turn might improve the robustness of the developed algorithm. All slides were digitized with a linear scanner (ScanScope CS2; Leica) in 1 focal plane at 400× magnification and a resolution of 0.25 µm per pixel. Focus points for scanning had to be selected manually for some slides to improve WSI quality. One slide stained with Prussian blue was excluded due to insufficient number of cells (< 300) present on the slide. In 12 WSIs stained with the modified Turnbull’s blue method, budding fungal hyphae and conidiophores were detected. The most reasonable explanation was fungal contamination of the staining solution. As fungal conidiophores may be difficult to distinguish from hemosiderophages with special iron staining, we excluded 2 WSIs with a proportion of > 1% of fungal conidiophores among alveolar macrophages. The other 10 cases had a proportion of conidiophores of < 1% and influence on the THS was considered negligible. Of the 3 excluded WSIs, the corresponding WSI of the same BALF sample with the other staining methods was also excluded for consistent statistical analysis. The final study set comprised 26 WSIs for each staining method (ie, a total of 52 WSIs).

### Annotators’ Scoring

**Figure 2.**
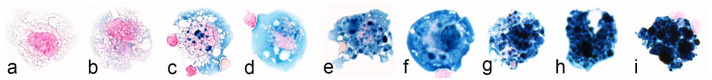
Schematic of the continuous hemosiderin content in alveolar macrophages stained with Perl’s Prussian blue. According to the scoring system by Doucet and Viel,^14^ macrophages have to be classified into 5 discrete grades, for which the cut-offs may be applied variably between different annotators. The following classification of the macrophages is according to the ground truth annotator. (a) Grade 0. (b) Borderline between Grades 0 and 1. (c) Grade 1. (d) Borderline between Grades 1 and 2. (e) Grade 2. (f) Borderline between Grades 2 and 3. (g) Grade 3. (h) Borderline between Grades 3 and 4. (i) Grade 4.

THSs of the 52 WSIs were performed by 10 annotators (J.S., F.B., J.B.M., A.K.B., G.B., M.E.G., A.G., K.d.P., K.W., and J.H.) including 5 board-certified veterinary clinical pathologists, 4 veterinary clinical or anatomic pathologists in training, and 1 equine internal medicine specialist with experience in equine BALF cytology. Participants were provided with the original publication on the THS system for horses^14^ and were instructed to follow that method. Hemosiderin content in macrophages is a continuum (Fig. 2) and it may be difficult to apply the thresholds between the 5 discrete grades as defined in the original study.^14^ Nevertheless, we decided against providing more detailed instructions with respect to the grading thresholds to keep the observer variability to a realistic degree of current diagnostic practice. To both view WSIs and label each cell included in the THS, the offline SlideRunner annotation software^3^ (*N* = 9) or the online annotation platform EXACT^21^ (*N* = 1) was used. Each annotators created a database containing centroid coordinate annotations (spot annotation in the middle of the cell) of the enumerated macrophages with individual label classes for the 5 hemosiderin grades. To ensure that annotators labeled at least 300 cells, we developed a plug-in software tool that automatically counted the annotations of all label classes combined and notified the annotator when 300 annotations had been made. Seven annotators measured the time required to perform the THS in each WSI. Time measurement started with labeling the first macrophage and ended after labeling the last macrophage.

### Supervised Deep Learning-Based Algorithms

For development of deep learning-based models using supervised learning, a state-of-the-art object detection network, RetinaNet, was used as previously described by Marzahl et al.^20^ The model was trained with reference annotations (ground truth dataset) for the 52 cases (see below). The cases of the ground truth dataset were split into 3 groups for 3-fold cross-validation. Three models were developed that each used a different subset of the data for training the model (training set), validating the training process (validation set), and testing the performance of the final model (test set). Thereby, we were able to analyze all 52 WSIs with our algorithms while avoiding testing algorithmic performance on the same images that were used for training or validation. To guarantee that all 3 subsets of the split dataset contained cases with Grade 4 cells, we sorted the cases by their number of grade 4 cells and assigned them in alternating order to these groups. The models were trained with the Adam optimizer and a maximal learning rate schedule of 0.001 until convergence was reached on the respective validation set (early stopping paradigm), as previously described.^20^

### Reference Methods

A true gold standard for quantification of hemosiderin in BALF is not available.^10,12,35^ For comparison of the performance of the THS determined by 10 annotators and the deep learning-based algorithm, we used different reference methods: (1) mean annotators’ THS, (2) ground truth THS, and (3) laboratory tests (RBC count, hemoglobin, and iron concentration). The human and algorithmic performance in assigning individual macrophages into the 5 hemosiderin grades was compared with the ground truth cell annotations.

#### Mean annotators’ THS

The mean THS of the 10 annotators was calculated for each WSI based on the ≥ 300 annotations of each annotator (see above). This reflects the consensus of the 10 annotators and thereby the difference (systematic error) between each annotator is averaged.

#### Ground truth annotations

The ground truth dataset used in this study contained annotations for all alveolar macrophages of the 52 WSIs. Annotations were created by 1 experienced annotator (CAB). The ground truth is a theoretical concept of “correct” annotations.^2^ However, errors in the ground truth cannot be avoided as it is created by an annotator. We have tried to mitigate human errors by applying a computer-assisted labeling approach. For the analysis of the study results, either labels of the hemosiderin grade per alveolar macrophage or the overall ground truth THS (score for all cells annotated in the slide) were used.

The used ground truth dataset has been published by Marzahl et al^23^ and detailed labeling methods and dataset description can be found in that paper. In summary, development of the final ground truth dataset was done in 5 consecutive steps: (1) expert-derived annotations of 16 WSIs, (2) development of a deep learning-based algorithm (based on the dataset from step 1), (3) creation of algorithm-derived annotations in the remaining 36 WSIs, (4) diligent review of the expert-derived and algorithm-derived annotations in all 52 WSIs, and (5) review of the assigned label classes assisted by a histogram-like clustering of all annotations. Grading of alveolar macrophages was done according to the definitions by Doucet and Viel.^14^ Algorithmic pre-annotations (step 3) of the 36 WSIs were performed to increase efficiency of dataset development (algorithm-expert collaboration). A previous study showed the suitability of this approach for dataset development.^22^ Histogram-like clustering of cell patches based on a continuous, algorithmic regression score (density maps) allowed review of the assigned grade and thereby improved consistency of assigning the discrete hemosiderin grades.

#### Laboratory evaluation of BALF

To obtain a more objective measure than the mean THS of the annotators or the ground truth THS, we measured other components of blood or its degradation products in BALF, ie, the RBC count, hemoglobin concentration, and iron concentration. Chemical quantification of hemosiderin is not possible. A 5-ml aliquot of BALF was analyzed for RBC and hemoglobin content using a CELL-DYN 3700 hematology analyzer (Abbott Laboratories, Abbott Park, IL, USA). RBC counts were obtained by means of impedance technology and hemoglobin determination was performed using the modified hemoglobin-hydroxylamine method. The remainder of the fluid aliquot was centrifuged and the cell-lysed supernatant used for iron determination. Iron was measured using an AU680 (Beckman Coulter, Inc., Brea, CA, USA) using a chromogenic method with reduction of iron, and subsequent formation of a complex of Fe^2+^ with 2,4,6-tri-(2-pyridil)-5-5triazine, which is measured photometrically.

### Statistical Analysis

All calculations were performed using R version 4.1.2 (R Foundation, Vienna, Austria). Diagnostic accuracy of EIPH based on the published diagnostic THS cut-off value of ≥ 75^14^ was calculated in comparison with the ground truth and the mean annotators’ score.

To calculate measures of variance including, overall measurement error, error between annotators and residual error, and the intraclass correlation coefficient (ICC) for the THS, a mixed model for a fully crossed, single measure, agreement design (ICC [was fitted using the R package lme4, version 1.1-27.1]).^5^ The percentage reduction of overall THS measurement error and reduction in between the annotators due to grade standardization was calculated by fitting 2 models using the score before and after standardization as outcome.

Cell-level color grading standardization was done by matching the selected cells of every annotator with the cells in the ground truth dataset or the algorithmic dataset, both of which aimed to contain annotations/predictions for every macrophage in the WSIs. Macrophage annotations were considered matching if the Euclidean distance between the center coordinates of both annotations was ≤ 50 pixels apart. Annotations without a match in the ground truth dataset were excluded from the standardized grading.

To evaluate the effect of the overall measurement error on the diagnosis of EIPH, we calculated an 80% uncertainty interval around the classification threshold of ≥ 75.^14^ This uncertainty interval, analogous to the definition of reference intervals,^15^ is defined as the 10% and 90% quantiles of the annotators’ measurement errors. For individual annotator scores within this interval, the probability that the diagnosis matches the diagnosis of the mean annotators’ score is less than 80% and thus should be considered unreliable and not reproducible.

The correlation between the individual annotators’ scores, the mean annotators’ score, the ground truth score, the algorithmic score, and the BALF RBC count, hemoglobin, and iron concentration was calculated using a Spearman correlation.

## Results

**Figure 3.**
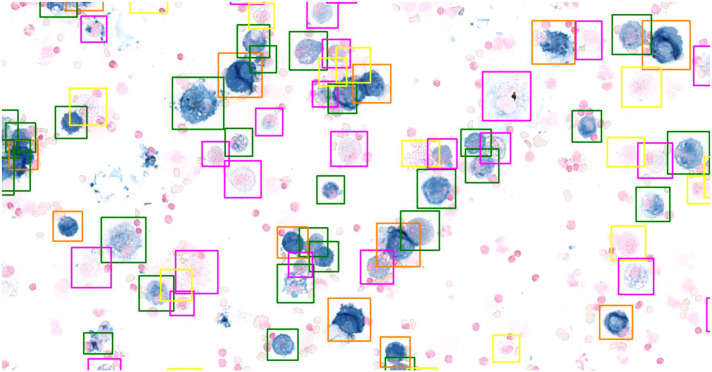
Cytologic image of a bronchoalveolar lavage fluid stained with Prussian blue. The boxes around the cells represent algorithmic detections of alveolar macrophages with assigned hemosiderin grades according to the scoring system by Doucet and Viel.^14^ Yellow box, hemosiderin grade 0; pink box, hemosiderin grade 1; green box, hemosiderin grade 2; orange box, hemosiderin grade 3; hemosiderin grade 4 not present in the image.

All 10 participants annotated at least 300 macrophages in each of the 52 WSIs, thereby creating 158,143 annotations. Annotators had different selection patterns of the 300 cells per image: While some annotators screened consecutive fields of view and annotated all macrophages within those fields, others screened the slide in a longitudinal or meandering pattern or selected evenly distributed image locations and annotated some macrophages within these fields. The ground truth dataset included all alveolar macrophages in the 52 WSIs, and consisted of 215,426 annotations (median: 4137 per slide; range: 596–8954 per slide). The deep learning-based algorithm analyzed the entire image of the 52 WSIs (Fig. 3) and detected 218,003 macrophages (median: 3943 per slide; range: 683–8670 per slide).

Time measurements for annotations were available for 7 annotators and 358 WSIs. The median time per case was 14:01 minutes for all annotators combined, and the median time per case ranged between 08:11 and 19:00 minutes for individual annotators. Automated analysis using the deep learning-based algorithms of the entire slides took 1:37 minutes on average (min: 1:31 minutes and max: 1:54 minutes) for each of the 52 WSIs using a modern graphics processing unit (NVIDIA P5000).

### Annotators’ THS: Consistency and Source of Error

**Figure 4.**
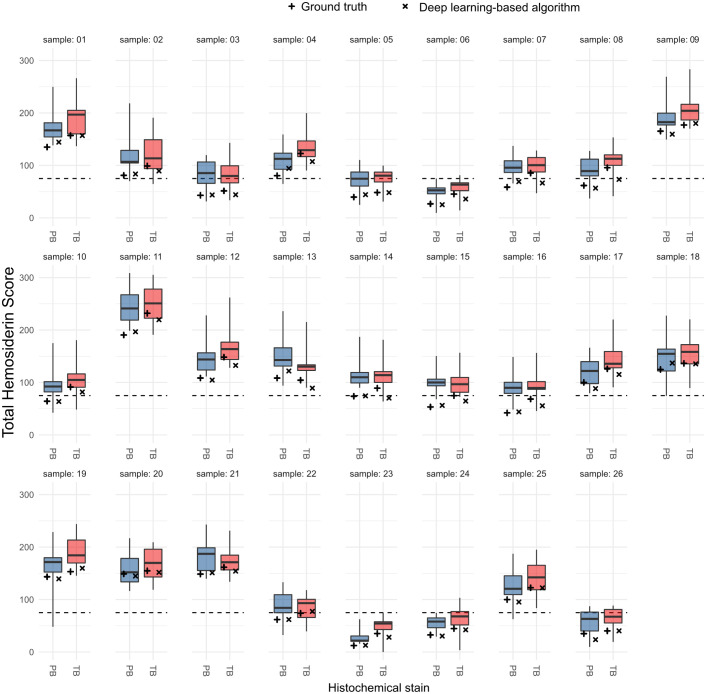
Comparison of the annotators’ total hemosiderin scores (box plots; *N* = 10) with the ground truth score (+) and the algorithmic score (×) separately for the 2 staining methods (blue boxplot: Prussian blue, PB; red boxplot: modified Turnbull’s blue, TB). Broken lines represent the cut-off value for diagnosis of exercise-induced pulmonary hemorrhage (EIPH; total hemosiderin score = 75) published by Doucet and Viel.^14^

The THS had notable variability between the 10 annotators (Fig. 4). The interquartile range of the difference to the mean annotators’ THS was 30 score points (–16 to +14) for all cases combined. The ICC for the THS of the 10 annotators was 0.685, ie, the scoring variance can be explained to 68.5% by a systematic error (difference between annotators in executing the THS) and to 31.5% by a random error (inconsistency within each annotator). Generally, the mean THS of the annotators was somewhat higher than the ground truth THS (on average 25.3 score points). Comparison of the 2 staining methods revealed that the THS determined from slides stained with modified Turnbull’s blue were higher than the THS from the corresponding slide stained with Prussian blue (on average 8.5 score points; standard error: 11.1). A stronger tendency of the THS difference due to the staining methods was observed in the ground truth dataset (average difference of 19.2 score points; standard error of 11.7).

**Figure 5.**
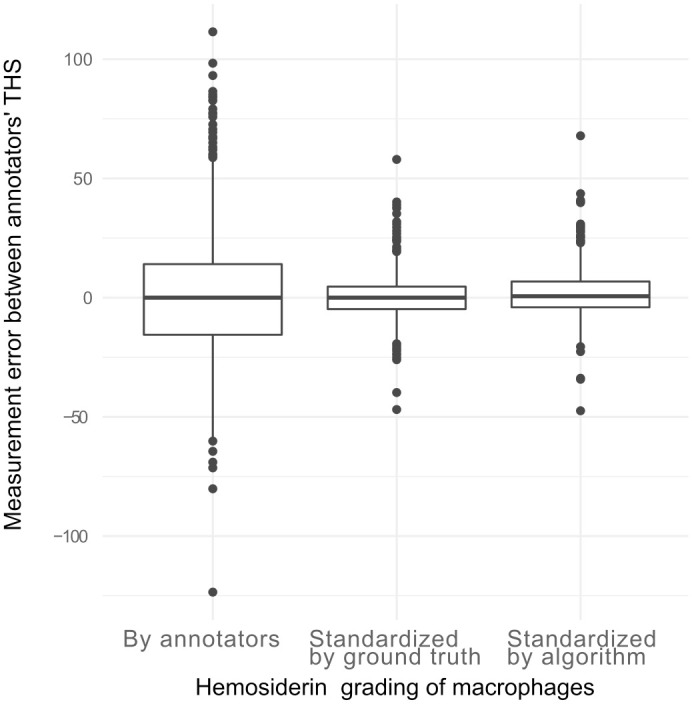
Comparison of the total hemosiderin score (THS) differences of individual annotators to the mean THSs of all annotators. For the left box plot, the hemosiderin grade of each included macrophage was derived from the annotators’ annotations. The measurement error is derived to a large proportion from interobserver variability in hemosiderin grading (systematic error). For the middle and right box plots, the cells selected by the annotators were graded according to the hemosiderin label of the ground truth annotations (middle boxplot) or the algorithmic predictions (right boxplot), thereby eliminating the interobserver variability of hemosiderin grading.

To evaluate the variability in hemosiderin grading, we compared the hemosiderin grade of each cell annotated by the annotators with the hemosiderin grade of the ground truth dataset. For the 158,143 annotations, we could find a cell-matched ground truth label in 121,217 (76.7%) cases. Only 61.7% (76,051/121,025) of the matched macrophage annotations had the same hemosiderin grade (Supplemental Table S1). Most of the divergent labels (93.6%, 42,411/44,974) differed only by one-grade level. The annotators assigned a higher grade in 37,919/44,974 divergent labels (84.3%). Subsequently, we exchanged the hemosiderin grade assigned by the annotators with the hemosiderin label from the ground truth dataset for all the matched cells, thereby creating a grade-standardized THS. Fig. 5 shows that the measurement error of the THS (difference between the annotators) was markedly reduced when using the standardized hemosiderin grade. Variance analysis determined that the overall measurement error was reduced by 87.8%. The systematic error of annotators was reduced by 97.7%, proving high variability between annotators in applying the published hemosiderin grade stratifications (ie, judging color saturation). The random error was reduced by 66.4% when the grade-standardized THS was used, which can be explained by the higher consistency in the ground truth dataset that was achieved by the multi-step labeling approach (see the “Materials and Methods” section).

### Algorithmic THS: Comparison with Annotators’ and Ground Truth Annotations

The algorithmic THS was generally lower than the mean annotators’ THS (on average 28.6 score points); however, it was similar to the ground truth THS (average difference of 3.2 score points; Fig. 4). The mean difference of the algorithmic THS between the 2 staining methods was 10.6 score points (standard error: 12.7). Algorithmically detected macrophages could be matched (Euclidean distance of ≤ 50 pixels) with 86.6% (186,650/218,003) of the ground truth annotations and 76.5% (121,025/158,143) of the participants’ annotations. Agreement between the assigned hemosiderin grade labels of these cell-matched annotations was much higher between the algorithm and the ground truth (accuracy: 91.3%; 170,322/186,650, Supplemental Table S2) than between the algorithm and annotators (62.8%; 76,051/121,025; Supplemental Table S3). Divergence between the algorithmic and ground truth hemosiderin grades, as well as the algorithmic and annotators’ hemosiderin grades differed mostly by one-grade level in 99.9% and 94.3% of instances, respectively. However, annotators had a clear tendency to assign higher grades than the algorithm. Of the cells with divergent hemosiderin grade labels, 84.3% (37,919/44,974) of the annotators’ labels were higher than the algorithmic label. In contrast, the divergent algorithmic labels had a higher grade level in 50.8% (8289/16,328) and lower grade level in 49.2% (8039/8289) of instances, as compared with the ground truth label. This explains why the algorithmic THSs are generally similar to the ground truth THSs, but notably lower than the annotators’ THSs.

### Diagnostic Accuracy of the Annotators’ and Algorithmic THS

In 28 of the 52 WSIs (54%), the THS value range of the 10 annotators overlaps with the diagnostic cut-off value (THS = 75); thus, there would have been inconsistencies in diagnosing EIPH between the 10 annotators (Fig. 4). Consensus on the EIPH diagnosis (THS above or below cut-off value) by 8/10 annotators was present in 82.7% of the cases (43/52, Supplemental Fig. S1) and consensus by 9/10 annotators was present in 69.2% of the cases (36/52; Supplemental Fig. S2 and Table S4). When using the grade-standardized THS of the annotators, the consensus for 9/10 annotators increased to 90.4%.

**Figure 6.**
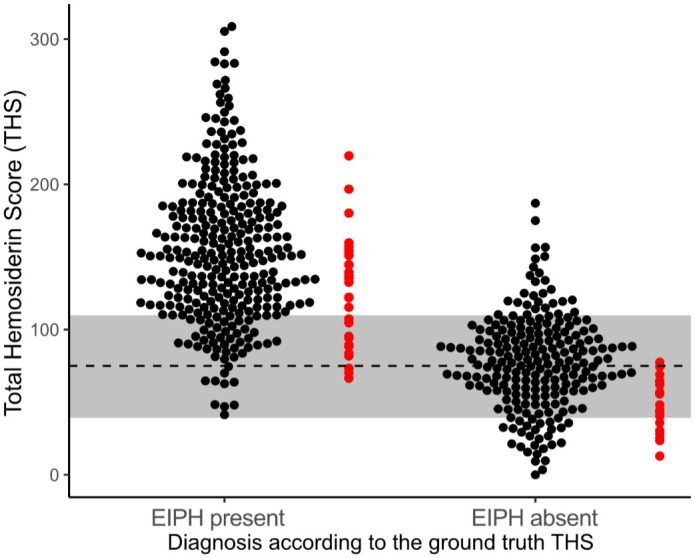
Scatter plots for the total hemosiderin scores (THSs) determined by the 10 annotators (black dots) and deep learning-based algorithm (red dots). The 52 cases are separated based on the ground truth THS being above or below the diagnostic cut-off value of 75 indicated by the broken line. The gray bar around the broken line is the diagnostic 80% uncertainty interval determined for the human annotators in this study.

Compared with the ground truth diagnosis of EIPH (ground truth THS < 75 or ≥ 75), annotators accurately classified the cases in 75.7% with a range of 63.5%–92.3% for individual annotators (Table 1, Fig. 6). The algorithmic THS had an accuracy of classifying the presence or absence of EIPH of 92.3%. When comparing the algorithmic and individual annotator’s THS with the mean annotators’ THS, diagnostic accuracy was higher for annotators (89.0%) than for the algorithmic approach (71.2%). When analyzing the mean annotators’ THS, the THS range for which there was less than 80% probability of being consistent with a diagnosis of EIPH was 39.2–109.8. For 58% instances of the annotators’ THSs that were within this range, the WSIs had not obtained consensus on a diagnosis of EIPH by 9/10 annotators, whereas, in 89% of instances with a THS outside (THS <39.2 or THS > 109.8) of this range, the WSIs had achieved consensus in 9/10 annotators.

**Table 1.** Diagnostic accuracy of exercise-induced pulmonary hemorrhage (EIPH) based on the total hemosiderin score (THS; above or below diagnostic cut-off of 75) of the 10 annotators and the deep learning-based algorithm.

	Accuracy for Diagnosis of EIPH (above or below cut-off)			
	Compared With the Ground Truth THS			Compared With the Mean Annotators’ THS
THS Method	All Cases (N = 52)	Cases Stained with Prussian Blue (*N* = 26)	Cases Stained with Modified Turnbull Blue (*N* = 26)	All Cases (*N* = 52)
All annotators combined	75.7%	69.6%	81.9%	89.0%
Individual annotators	63.5%–92.3% (median: 75.0%)	57.7%–84.6% (median: 69.2%)	69.2%–100% (median: 80.8%)	60.8%–98.1% (median: 92.3%)
Algorithm	92.3%	100%	84.6%	71.2%

### Correlation With the Reference Methods

The mean annotators’ THSs and algorithmic THSs had very high correlations (*R* = 0.98) with the ground truth THSs (Table 2). The same correlation (*R* = 0.98) was identified when the algorithmic THSs were compared with the mean annotators’ THS, whereas, individual annotators had a correlation of 0.94 to 0.97 to the mean annotators’ THS.

To facilitate an annotator-independent evaluation of the 3 total hemosiderin scoring methods (the annotators’ THS, the ground truth THS, and the algorithmic THS), each method was correlated with the RBC count and chemical measurements of hemoglobin and iron concentration. The RBC count and hemoglobin concentration did not correlate with any THS method (Table 2). The algorithmic THS had a slightly higher correlation with the iron measurement (*r* = 0.79) than the individual (*r* = 0.61–0.79) or mean annotators’ (*r* = 0.75) THS or ground truth THS (*r* = 0.75). The iron concentration ranged between < 0.4 and 4.7 µmol/L and was below the measurable threshold (< 0.4 µmol/L) in 9 cases.

**Table 2.** Spearman’s correlation of the annotator’s, ground truth, and algorithmic total hemosiderin score (THS) with the ground truth THS as well as the red blood cell count, hemoglobin concentration, and iron concentration from bronchoalveolar lavage fluid.

THS Method	Ground Truth THS	Red Blood Cell Count	Hemoglobin Concentration	Iron Concentration
Mean annotators’ THS	.98	.08	.11	.75
THS of individual annotators	.91–.97 (median: .94)	−.11 to .14 (median: .05)	−.07 to .19 (median: .11)	.61–.79 (median: .72)
Ground truth THS	1.0	.06	.15	.75
Algorithmic THS	.98	.12	.20	.79

## Discussion

The THS by Doucet and Viel^14^ is considered to be one of the most sensitive and accurate tools for the diagnosis of EIPH. However, this method is regarded as too time-consuming for a routine diagnostic test^10^ and it has not been used in prevalence studies to screen large horse populations. In this study, we evaluated automated image analysis as an approach to improve speed, accuracy, and reproducibility of the THS. Our algorithm was able to score thousands of cells in less than 2 minutes and had equivalent diagnostic accuracy compared with the annotators.

In this study, we also evaluated interobserver variability of the THS and showed that there is high systematic error between annotators. Variability between the annotators’ THSs might have resulted from the following sources: (1) bias in selection of the 300 macrophages (representativeness of the included cells) and (2) variability and inconsistency in grading the intracytoplasmatic hemosiderin content of each cell. Regarding the first source of variability, we noticed that the annotators had different selection patterns, which did not seem to have an obvious influence on the annotator’s variability. We determined that many of the expert’s annotations could not be matched with the ground truth annotations or algorithmic predictions. The most likely explanation for this is that it is quite difficult to distinguish the different cell types using the special iron stain. The study annotators and ground truth annotator seemed to have difficulty to differentiate alveolar macrophages from the other cell types. However, most of the systematic errors arose from the differences in applying the hemosiderin grading stratification to alveolar macrophages. Inconsistency in hemosiderin grading was most relevant in cases that were close to the diagnostic cut-off, and led to a lack of consensus by the majority of annotators for the diagnosis of EIPH. For scoring by human experts, we therefore propose to use an 80% uncertainty interval of ± 35 score points around the published cut-off value of 75, for which the diagnosis of EIPH is not reproducible by a human expert. Annotators with THS values within this uncertainty interval, ie, THS values between 40 and 110, had a likelihood of a discrepant diagnosis in more than 20% of cases, when compared with the other annotators, for the presence or absence of EIPH. Our results highlight that increased standardization or specific training in the application of the scoring system is needed for future studies and for its use in the diagnostic setting. Development of a standardized “color chart” with images of alveolar macrophages with continuously increasing hemosiderin content and clearly defined thresholds might improve grading consistency between human experts. In our study, we determined that the hemosiderin grading of experts can be standardized by using algorithmic grade predictions, as this led to a marked reduction in the systematic error between annotators.

The present study identified that deep learning-based algorithms are able to achieve high performance for scoring hemosiderophages that was in many aspects equivalent to the performance of experts. However, a major limitation of the present study is the lack of a true gold standard^10,12,35^ to compare the performance of expert annotators with the performance of the deep learning-based algorithm without bias. For the development of deep learning-based algorithms for histopathological and cytological tasks, it is often the gold standard to compare the algorithmic predictions with expert-derived ground truth annotations.^2,6,20,25^ Nevertheless, it needs to be acknowledged that human errors in the ground truth labeling may have a bias on performance evaluation. This is why we sought to mitigate human errors in the ground truth dataset by using a multi-step, computer-assisted labeling approach,^23^ which our research group validated for this specific task in previous studies.^22^ As this ground truth dataset was also used to train the algorithmic models (using a 3-fold cross-validation), we found very high consistency between the ground truth dataset and algorithmic predictions, indicating that the models replicated the training data very well. When the ground truth THSs were used as the reference, the algorithm had a higher diagnostic accuracy than the annotators. In contrast, the 10 annotators had the clear tendency to assign higher hemosiderin grades to alveolar macrophages, ie, systematically applied lower thresholds for the individual hemosiderin grades than the ground truth annotator and algorithm. This explains the marked difference between the THSs of the experts and the ground truth and algorithmic individual THSs as well as the higher diagnostic accuracy of the individual annotators’ THSs compared with the mean annotators’ THS. Depending on the training data, the deep learning model can learn any desired threshold between the 5 cell grades. Adaptation of the algorithmic results to individual users by applying multiplication factors to the predicted cell classes or THS may not be desired, as the scoring methods should be keep consistent between pathologists and laboratories.

Due to the above mentioned bias of the mean annotators’ THS and ground truth dataset as a reference method, we evaluated 3 laboratory tests, which are observer-independent (RBC count, and hemoglobin and iron concentrations in BALF). We found that the iron concentration had a high correlation with the THSs, whereas, the RBC count and hemoglobin concentration did not correlate with the THSs. RBCs and hemoglobin are features of acute pulmonary bleeding and are degraded shortly after the hemorrhagic event and therefore seem to be inappropriate reference methods for the THS, which measures chronic hemorrhage. In the present study, we used iron concentration for the first time as a measure of pulmonary bleeding. The limitation of the chemical iron measurement was the low iron content in BALF, which in some cases was below the limit of detection. Future studies are needed to determine the value of iron concentration as a reference method for the THS and as a potential diagnostic test for EIPH. Potential source of bias of the iron concentration is the variable cell density in BALF (and thus variable density of alveolar macrophages) and contamination with RBC.

Automated image analysis using deep learning is a highly relevant evolving technique, used in various fields of research in veterinary clinical, anatomic, and toxicologic pathology. Algorithms are mainly applied with the goal to increasing accuracy, reproducibility, and time efficiency of quantitative tasks.^1,6,8,24,28,36^ A precondition for computerized analysis is the availability of digital images, which is aided by the current trend of digitizing the diagnostic workflow of pathology laboratories. The use of digital microscopy for cytological specimens is, however, hampered by limited image resolution and lack of fine focus of default WSIs.^7,8^ Nevertheless, the annotators of the present study consider WSIs appropriate to perform the THS in this study’s cases, because of the uniform depth of the samples, and as relatively little cellular detail is necessary to evaluate the intracytoplasmic hemosiderin content of macrophages. Another limitation of WSIs specific to the task of scoring hemosiderophages is that different WSI scanners often exhibit a marked difference in the color representation, ie, they might have a higher or lower intensity of the blue color. This is likely to influence annotators and algorithms in evaluating the amount of blue pigment and needs to be evaluated in future studies. Currently, there are few studies that have evaluated the benefits of automated image analysis compared with the visual assessment by experts in veterinary medicine.^4,6,9,20^ These studies are needed to critically evaluate potential sources of algorithmic errors before an algorithm can be used for routine diagnostic purposes. Based on our results, we suggest that algorithms may improve accuracy, reproducibility, and time efficiency and are therefore potentially useful for a routine diagnostic or research setting. Future studies need to evaluate how THS algorithms are best implemented in a diagnostic workflow, while ensuring high diagnostic reliability. Generally, algorithms can be used to automatically predict the diagnosis or they can be used as an assistive tool, which supports annotators in critical steps of the diagnostic task (computer-assisted diagnosis). For the diagnosis of EIPH, THSs could be derived fully automatically by the algorithm with only rough verification of the predictions by an expert. Alternatively, algorithms could be used to standardize hemosiderin grading of individual macrophages that are selected by annotators (computer-assisted THS). The benefits of both applications on diagnostic accuracy and reproducibility have been demonstrated in this study.

## Conclusion

Cytologic quantification of hemosiderin content in alveolar macrophages using the THS is considered the most sensitive method for the diagnosis of EIPH. However, we have shown that the THS by human experts is time-consuming and there is high interobserver variability (systematic error) in applying the scoring criteria. We propose to use an uncertainty interval of 75 ± 35 score points for the diagnosis of EIPH by experts. Furthermore, to overcome the limitations of human experts, we validated a deep learning-based image analysis algorithm that had high accuracy/correlation compared with the mean THSs of 10 annotators, a ground truth dataset, and iron concentrations of BALF. We have shown that deep learning-based algorithms are a valuable tool for time-efficient, accurate, and reproducible scoring of hemosiderophages, which could be applied to research studies, such as large prevalence studies and routine diagnostic service.

## Supplemental Material

sj-pdf-1-vet-10.1177_03009858221137582 – Supplemental material for Cytologic scoring of equine exercise-induced pulmonary hemorrhage: Performance of human experts and a deep learning-based algorithm Click here for additional data file. Supplemental material, sj-pdf-1-vet-10.1177_03009858221137582 for Cytologic scoring of equine exercise-induced pulmonary hemorrhage: Performance of human experts and a deep learning-based algorithm by Christof A. Bertram, Christian Marzahl, Alexander Bartel, Jason Stayt, Federico Bonsembiante, Janet Beeler-Marfisi, Ann K. Barton, Ginevra Brocca, Maria E. Gelain, Agnes Gläsel, Kelly du Preez, Kristina Weiler, Christiane Weissenbacher-Lang, Katharina Breininger, Marc Aubreville, Andreas Maier, Robert Klopfleisch and Jenny Hill in Veterinary Pathology
