# Supplementary material for: Cytologic scoring of equine exercise-induced pulmonary hemorrhage: Performance of human experts and a deep learning-based algorithm
Source: Vet Pathol. 2022 Nov 17;60(1):75–85. doi: 10.1177/03009858221137582 (PMC9827485; doi:10.1177/03009858221137582)
Supplement: sj-pdf-1-vet-10.1177_03009858221137582 – Supplemental material for Cytologic scoring of equine exercise-induced pulmonary hemorrhage: Performance of human experts and a deep learning-based algorithm [file sj-pdf-1-vet-10.1177_03009858221137582.pdf]

Supplemental Material

Cytologic Scoring of Equine Exercise-Induced Pulmonary Hemorrhage: Performance of Human Experts and a Deep Learning-Based Algorithm

Christof A. Bertram [1,2*], Christian Marzahl [3,4*], Alexander Bartel [2*], Jason Stayt [5], Federico Bonsembiante [6], Janet Beeler-Marfisi [7], Ann K. Barton [2], Ginevra Brocca [6], Maria E. Gelain [6], Agnes Gläsel [8], Kelly du Preez [9], Kristina Weiler [8], Christiane Weissenbacher-Lang [1], Katharina Breininger [3], Marc Aubreville [10], Andreas Maier [3], Robert Klopfleisch [2] and Jenny Hill [5]

Supplemental Table S1. Confusion matrix of the hemosiderin grades of alveolar macrophages assigned to the same cells by the ten annotators and the ground truth annotations.

		Ground truth's cell grade				
		0	1	2	3	4
Annotators' cell grade	0	28,646	2,448	87	16	0
	1	11,668	25,385	2,821	53	8
	2	486	15,215	15,213	1,784	5
	3	9	1,147	7,102	4,503	160
	4	4	71	1,051	2,275	1,060

For the 158,143 annotations made by the annotators, a match was found in the ground truth dataset (Euclidean distance between the annotators' and ground truth annotation of ≤ 50 pixels) in 121,217 (76.7%) instances. Of the 121,217 alveolar macrophages, 74,807 (61.71%) were assigned the same hemosiderin grade by the annotators and the ground truth. Of the 46,410 macrophages with divergent hemosiderin grade, 43,473 (93.67%) had a divergence by one grade level, 2,829 (6.10%) had a divergence by two grade levels, 104 (0.22%) had a divergence by three grade levels, and 4 (<0.01%) had a divergence by four grade levels.

Supplemental Table S2. Confusion matrix of the hemosiderin grades of alveolar macrophages assigned to the same cells by the ground truth annotator and the deep learning-based algorithm.

		Ground truth‘s cell grade				
		0	1	2	3	4
Algorithmic cell grade	0	58,341	2,934	3	1	0
	1	2,413	62,627	2,867	0	0
	2	10	4,347	36,516	1,971	0
	3	3	4	1,326	11,615	263
	4	1	0	1	184	1,223

For the 218,003 algorithmic predictions, a match (Euclidean distance between the pathologist’s and ground truth annotation of ≤ 50 pixels) was found in the ground truth dataset in 186,650 (85.62%) instances. Of the 186,650 alveolar macrophages, 170,322 (91.25%) were assigned the same hemosiderin score by the ground truth annotator and the algorithm. Of the 16,328 macrophages with divergent hemosiderin grade, 16,305 (99.86%) had a divergence by one grade level, 18 (0.11%) had a divergence by two grade levels, 4 (0.02%) had a divergence by three grade levels, and 1 (<0.01%) had a divergence by four grade levels.

Supplemental Table S3. Confusion matrix of the hemosiderin grades of alveolar macrophages assigned to the same cells by the ten annotators and the deep learning-based algorithm.

		Algorithmic cell grade				
		0	1	2	3	4
Annotators‘ cell grade	0	30,228	2,301	73	18	0
	1	11,595	24,986	2,911	44	5
	2	426	14,378	15,603	1,530	5
	3	10	918	7,279	4,237	168
	4	2	51	1,011	2,249	997

For the 158,143 annotations made by the annotators, a match (Euclidean distance between the annotators' and ground truth annotation of ≤ 50 pixels) was found in the algorithmic predictions in 121,025 (76.53%) instances. Of the 121,025 alveolar macrophages, 76,051 (62.84%) were assigned the same hemosiderin grade by the annotators and the algorithm. Of the 44,974 macrophages with divergent hemosiderin grade, 42,411 (94.30%) had a divergence by one grade level, 2,477 (5.51%) had a divergence by two grade levels, 84 (0.19%) had a divergence by three score levels, and 2 (<0.01%) had a divergence by four grade levels.

Supplemental Table S4. Degree of consensus by the ten annotators on exercise-induced pulmonary hemorrhage diagnosis (THS score above or below 75).

Consensus definition	Number of cases with consensus	
	Annotator's THS	grade-standardized THS
By all of the 10 annotators	24 / 52 (46%)	40 / 52 (77%)
By at least 9/10 annotators	36 / 52 (69%)	47 / 52 (90%)
By at least 8/10 annotators	43 / 52 (83%)	50 / 52 (96%)
By at least 7/10 annotators	49 / 52 (94%)	52 / 52 (100%)
By at least 6/10 annotators	50 / 52 (96%)	52 / 52 (100%)
By at least 5/10 annotators	52 / 52 (100%)	52 / 52 (100%)

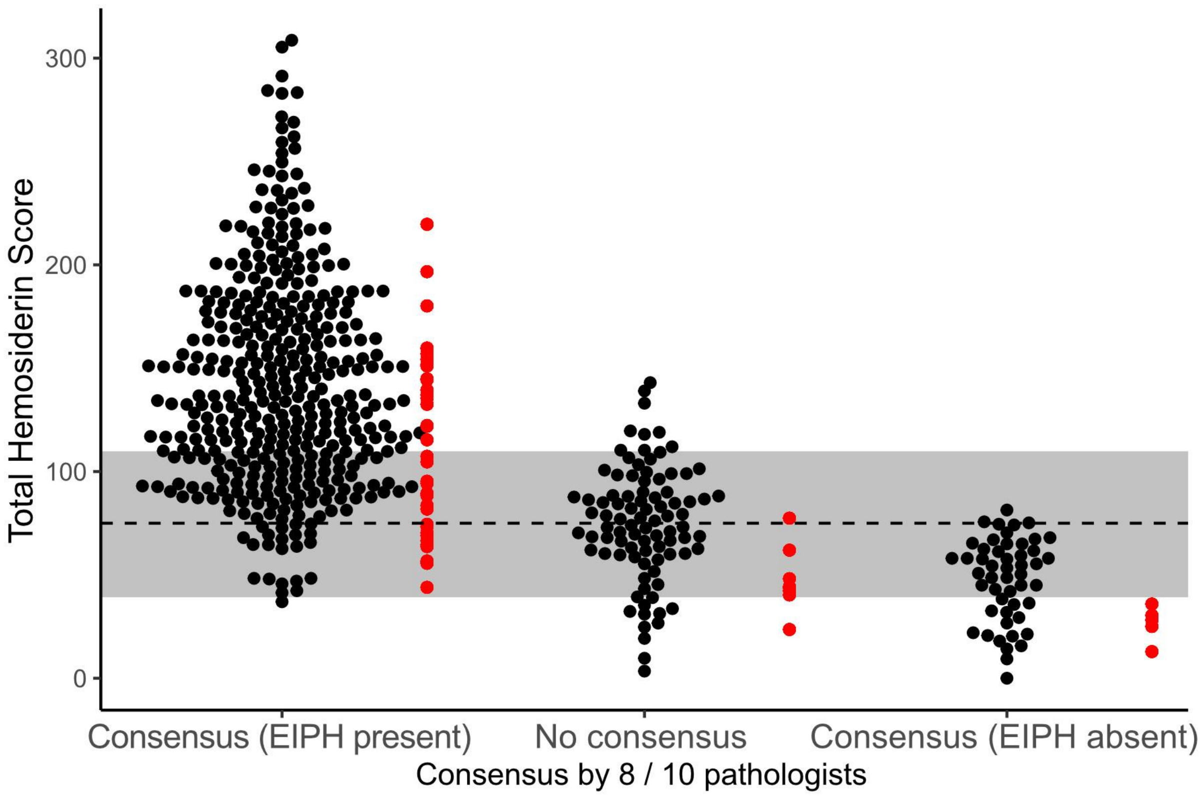

Supplemental Fig. S1. Scatter plots for total hemosiderin scores (THSs) determined by the ten annotators (black dots) and deep learning-based algorithm (red dots). The 52 cases are separated based on their consensus of the exercise-induced pulmonary hemorrhage (EIPH) diagnosis by 8 out of 10 annotators. The left scatter plot represents the THS values for cases with a consensus on THS values above the cut-off of 75 (N = 32), the middle scatter plot represents the THS values for cases with no consensus (N = 16) and the right scatter plot represents the THS values for cases with a consensus on THS values below the cut-off of 75 (N = 4). The broken line indicates the diagnostic cut-off at a THS of 75 and the grey bar around the broken line is the reference range determined in this study.

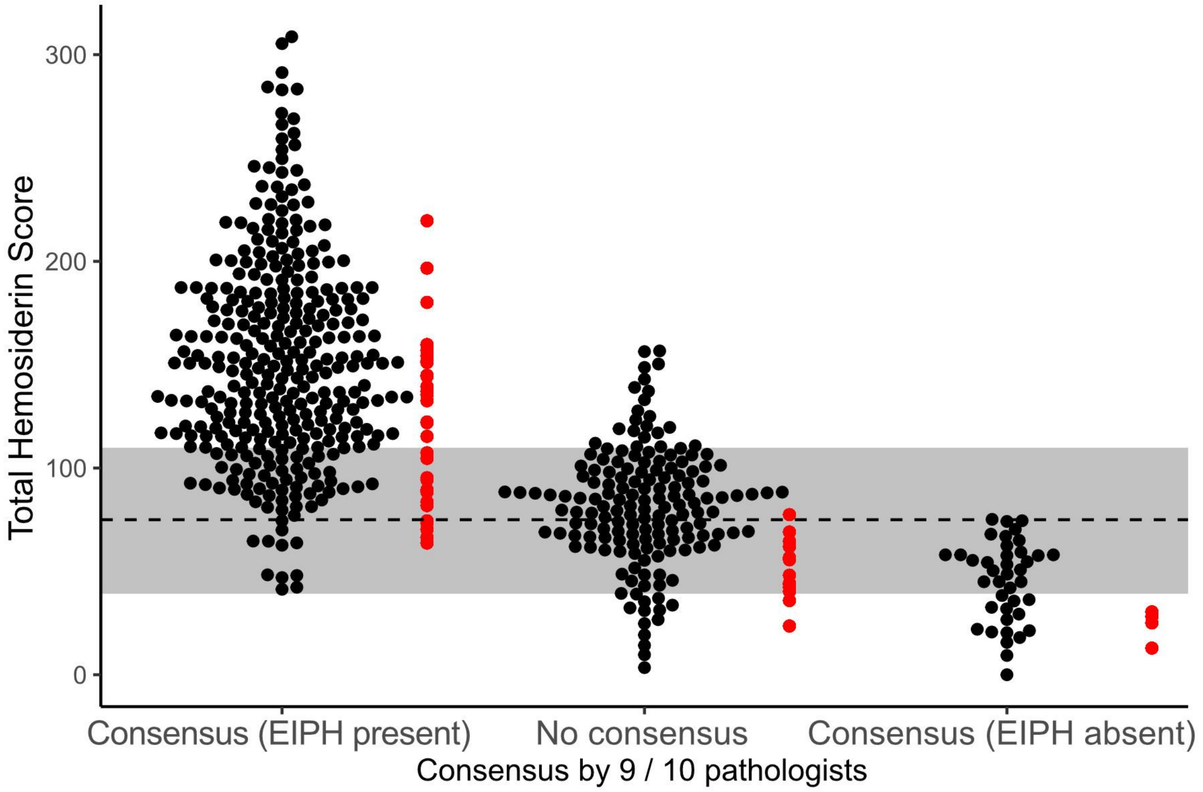

Supplemental Fig. S2. Scatter plots for total hemosiderin scores (THSs) determined by the ten annotators (black dots) and deep learning-based algorithm (red dots). The 52 cases are separated based on their consensus of the exercise-induced pulmonary hemorrhage (EIPH) diagnosis by 9 out of 10 annotators. The left scatter plot represents the THS values for cases with a consensus on THS values above the cut-off of 75 (N = 32), the middle scatter plot represents the THS values for cases with no consensus (N = 16) and the right scatter plot represents the THS values for cases with a consensus on THS values below the cut-off of 75 (N = 4). The broken line indicates the diagnostic cut-off at a THS of 75 and the grey bar around the broken line is the reference range determined in this study.
